# Supplementary material for: SUBATOMIC: a SUbgraph BAsed mulTi-OMIcs clustering framework to analyze integrated multi-edge networks
Source: BMC Bioinformatics. 2022 Sep 5;23:363. doi: 10.1186/s12859-022-04908-3 (PMC9442970; doi:10.1186/s12859-022-04908-3)
Supplement: Supplementary file 7 — Additional file 7: Pipeline description and module stability. [file 12859_2022_4908_MOESM7_ESM.docx]

**Additional file 7**

This file contains a technical description of the pipeline implementation as well as details of the stability analysis.

**The directed acyclic graph of SUBATOMIC**


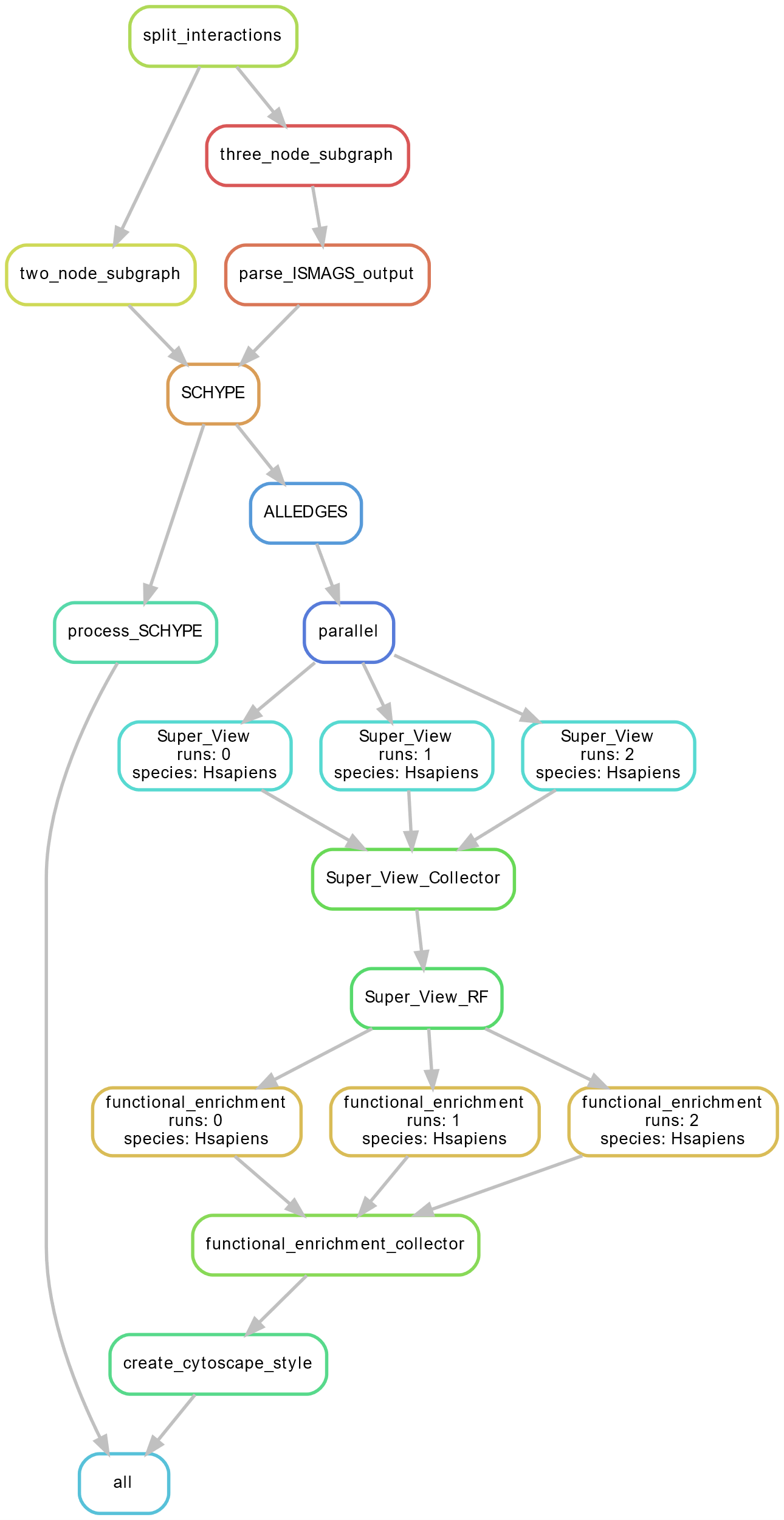


Figure 1: The directed acyclic graph (DAG) as implemented in SUBATOMIC for a run that utilizes three cores.

Figure 1 shows the directed acyclic graph (DAG) of SUBATOMIC for an example run that utilizes three cores. The rule ‘**split interactions**’ takes as input the interaction file with all edges and network letters and creates one formatted file for each network with a specific file naming. Then the graph splits into two branches: the rule ‘**two_node_subgraph**’ utilizes a custom python script to find two-node subgraphs, and the rule ‘**three_node_subgraph**’ utilizes ISMAGS to search for three-node subgraphs. The latter is further processed by the rule ‘**parse_ISMAGS_output**’ to bring the detected subgraphs in the right format for consecutive steps. The rule ‘**SCHYPE**’ preprocesses the input interaction files as well as the two- and three-node subgraphs and forms a hyper-edge network that is consequently used for clustering with SCHYPE. The graphs then branch again to process the SCHYPE output. The rule ‘**process_SCHYPE’** creates different module file formats (i.a. network file formats that can be imported into Cytoscape). Moreover, this process annotates module genes according to a user-defined functional annotation file (assign gene names for an identifier, add short functional description, classify gene as TF, miRNA or gene) and calculates the clustering coefficient for each module. The rule ‘**ALLEDGES**’ is another parser that brings the clustered modules in a format for the superview analysis. The rule ‘**parallel**’ splits up the set of all modules according to the number of available cores. This spawns a parallel execution of the next rule, ‘**Super_View**’ that calculates the superview for each module. The next rule ‘**Super_View_Collector**’ consolidates all results of the different parallel executions into single files for each module type. The rule **Super_View_RF**’ calculates the superview relations between all defined TF and miRNA regulators and modules. Consecutively, parallel executions of the rule ‘**functional enrichment**’ are spawned that use GOATOOLS for GO term enrichment analysis. Moreover, it calculates extra columns in the enrichment file such as fold change and p-value rank. The results of these runs are consolidated by the rule ‘**functional_enrichment_collector**’. The rule ‘**create Cytoscape style**’ modifies a pre-set Cytoscape style sheet and adapts it to the user-defined network letter representations and functional characterizations. The pipeline terminates when the ‘**all**’ rule is executed.

**Module stability based on re-sampling**

The stability prediction can be found in the result section of the main manuscript. Here, we present the boxplot and violin plot for two additional similarity metrics (AMI: Figure 2, JI: Figure 3).
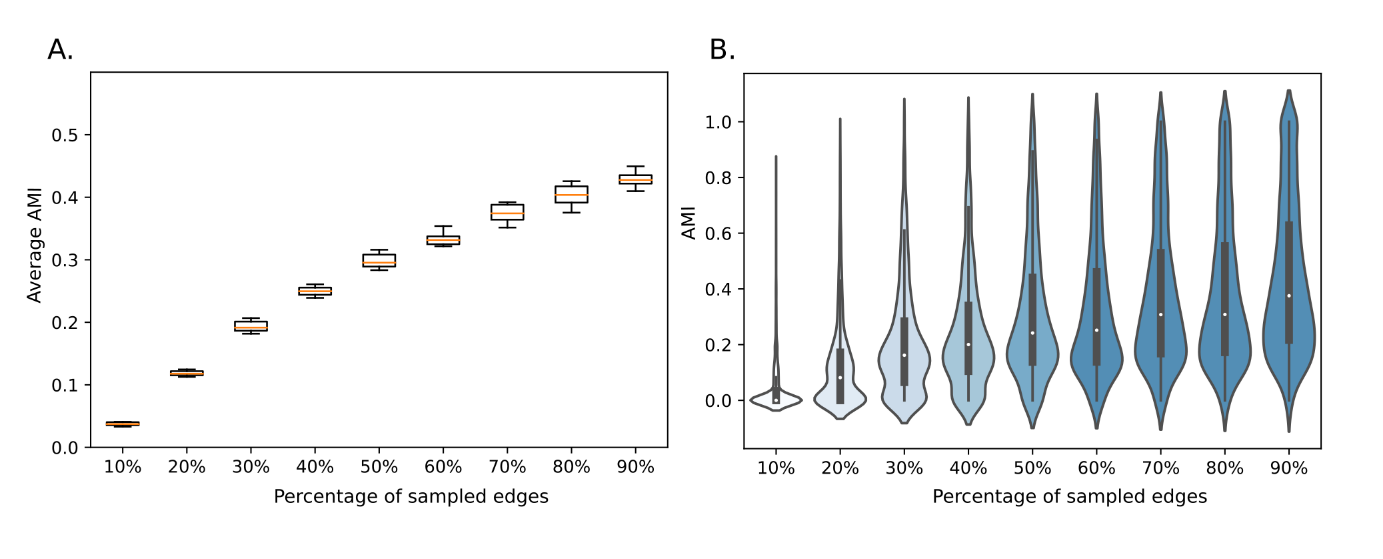


Figure 2 A: Boxplots representing the average Adjusted Mutual information score (AMI) for each subsampled set. Each box summarizes the result of 10 independent SUBATOMIC runs per 10%, 20%, … 90% of interactions sampled from the full network. The orange line inside each box represents the average AMI of the 10 repetitions. B: Violin plot representing the distribution of one selected run. The width of each violin indicates how many values were present for a certain AMI value.


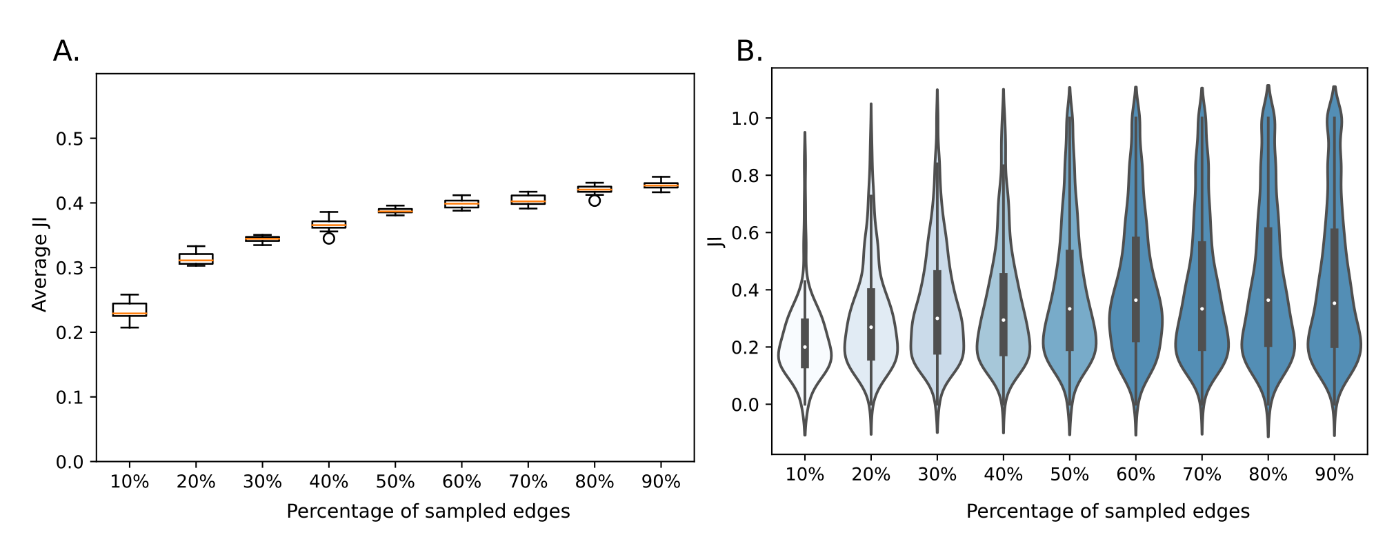


Figure 3 A: Boxplots representing the average Jaccard Index (JI) for each subsampled set. Each box summarizes the result of 10 independent SUBATOMIC runs per 10%, 20%, … 90% of interactions sampled from the full network. The orange line inside each box represents the average AMI of the 10 repetitions. B: Violin plot representing the distribution of one selected run. The width of each violin indicates how many values were present for a certain JI value.

**Module stability based on nPCC**

Given the hypoxia dataset of our method, we calculated the nPCC value for each of the modules. This value represents the pairwise average Pearson correlation in a module that was compared against a set of random modules to infer z-scores, which in turn were evaluated against a normal distribution to derive a p-value (see Methods). We could show that even for a phenotype specific expression dataset like hypoxia, 24% of all modules have a significant nPCC value (given a p-value > 0.05). This is another way of demonstrating that the derived modules are biologically coherent. Figure 4 shows the distribution of p-values for nPCC values in the dataset. The dataset was filtered for modules with at least 80% of their genes having an expression value in the hypoxia dataset.


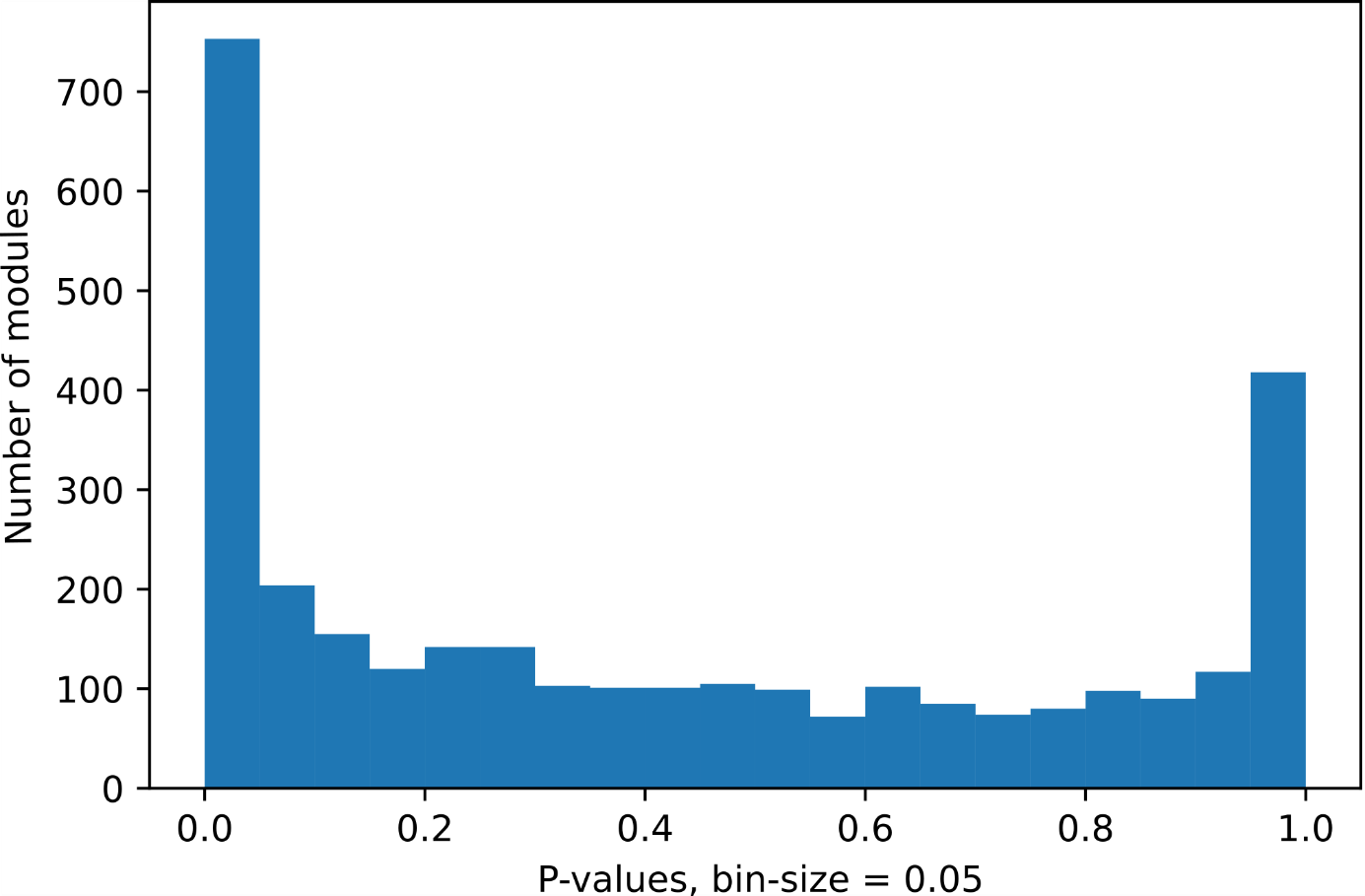


Figure 4: Distribution of p-values of nPCC values for modules with expression values available for at least 80% of the module genes. A total of 24% of all modules contained significantly higher correlated expression values than expected by chance (for p<=0.05).
